# Can emphysema influence size discrepancy between radiologic and pathologic size measurement in subsolid lung adenocarcinomas?

**DOI:** 10.1111/1759-7714.13165

**Published:** 2019-08-12

**Authors:** Jae‐Kwang Lim, Kyung Min Shin, Sang Yub Lee, Hoseok Lee, Myong Hun Hahm, Jaehee Lee, Chang Ho Kim, Seung‐Ick Cha, Ji Yun Jeong

**Affiliations:** ^1^ Department of Radiology School of Medicine, Kyungpook National University Daegu South Korea; ^2^ Department of Internal Medicine School of Medicine, Kyungpook National University Daegu South Korea; ^3^ Department of Pathology, School of Medicine, Kyungpook National University Daegu South Korea

**Keywords:** Emphysema, lung adenocarcinoma, size measurement, subsolid nodule

## Abstract

**Background:**

To investigate the difference in the measured diameter of subsolid lung adenocarcinomas of thin‐section computed tomography (TSCT) and pathology according to presence of emphysema.

**Methods:**

A total of 268 surgically resected pathologic T1 or T2 adenocarcinomas visualized as subsolid nodules (SSNs) on TSCT were analyzed in 252 patients. Two observers measured the greatest diameters of the whole tumor (WTsize) and solid tumor (STsize) on TSCT in lung windows, classified nodules as part‐solid or nonsolid, and recorded the presence of regional emphysema. Interobserver variability was determined with intraclass correlation coefficients (ICC). CT measurements were compared to pathologic size (Psize) and invasive size (PIsize) using the Wilcoxon signed‐rank test.

**Results:**

The interobserver agreement between the diameters measured by the two observers was strong for WTsize (ICC = 0.968 [95% confidence interval, 0.960–0.975]) and STsize (ICC = 0.966 [95% CI, 0.950–0.969]). Radiologic WTsize was significantly greater than Psize *(P* < 0.001), while STsize was less than PIsize. The WTsize of the emphysema group was better correlated with Psize than WTsize of the normal lung group (*P* = 0.001), while the STsize of the normal lung group was better correlated with PIsize than STsize of the emphysema group. The concordance rate in T staging between CT and pathologic analysis was better correlated in patients with normal lungs than in those with emphysema (*P* = 0.023).

**Conclusion:**

STsize on TSCT was underestimated in patients with emphysema, resulting in higher discordance in T staging between TSCT and pathologic analysis for subsolid lung adenocarcinomas.

## Introduction

Lung cancer is the leading cause of cancer death worldwide. Staging of lung cancer is crucial in correctly allocating patients to proper treatment and determining prognosis. Tumor size is a key parameter in TNM staging of lung cancer, which has been shown to be an independent predictor of survival in large databases, such as those assembled by the National Cancer Institute's Surveillance, Epidemiology, End Results registry, and the International Association for the Study of Lung Cancer (IASLC).^1^ Furthermore, the new eighth edition of the TNM classification of lung cancer revised by the American Joint Committee on Cancer (AJCC) subdivided the T descriptor for smaller lung cancers,^2^, ^3^ reflecting the importance of precise size measurement, as small differences in maximum tumor dimension play important roles in the prognosis of lung cancer.

According to previous studies, there may be size discrepancies between computed tomographic (CT) and pathologic measurements of lung cancer. For example, several studies have shown that CT measurement overestimate the pathologic size of the tumors.^4^, ^5^, ^6^ A possible explanation for these differences is that the inflation state of lung tissues during CT is different from that of the deflated lung tissue of resected specimens that shrink after fixation. The discrepancy in tumor size between CT and pathologic measurements was particularly greater in predominantly lepidic or ground‐glass lung adenocarcinomas (LACs),^7^, ^8^ suggesting that the ground‐glass component is more susceptible to inflation than the solid component. Therefore, it may be speculated that the measurement of a subsolid nodule (SSN) could potentially be affected by the presence of emphysema, since emphysema involves histologically overinflated air spaces due to irreversible disruption of the alveolar septa.

Goo *et al*. previously reported that regional emphysema surrounding a nodule had a significant effect on volume measurement of the solid nodule.^9^ However, to date, there have been no studies regarding the effect of emphysema on the size discrepancy between CT and pathologic measurements. Therefore, the purpose of the present study was to investigate whether the presence of emphysema could influence the discrepancy between CT and pathologic measurements in LACs presenting as SSNs.

## Methods

Our institutional review board approved this retrospective study and provided a waiver for informed consent.

### Patients

Using our hospital electronic medical record system, we retrieved a list of all patients who underwent surgical resection for LACs at Kyungpook National University Chilgok Hospital between January 2014 and September 2017. A total of 606 patients with surgically‐resected LACs were identified, of whom, 507 patients with LACs of pathological stage T1 or T2 were included in this study. Specimens with findings of atypical adenomatous hyperplasia or adenocarcinoma in situ on pathologic analysis were excluded. Data of patients meeting the initial inclusion criteria were then matched with the data in our institutional radiology database to identify patients whose pre‐resection CT examination images were available in the picture archiving and communication system (PACS) of our department. CT examinations of all 507 LACs were reviewed by two thoracic radiologists (J.K.L. and K.M.S., with seven and 11 years of experience in chest radiology, respectively) using the inclusion and exclusion criteria. This consensus review determined that 396 of 507 (78.1%) LACs manifested as subsolid nodules on CT. We excluded 60 patients who did not undergo preresection CT within 60 days before surgery, 45 patients for whom we were unable to obtain thin‐section CT (TSCT), five patients without adequate pathologic size measurements, 12 patients with inadequate CT images because of motion artifacts or insufficient inspiration, and six patients who underwent preoperative neoadjuvant chemotherapy. A total of 233 patients had a single SSN, while 13 patients had two, and five patients had three; thus, a total of 268 SSNs in 252 patients were analyzed in the present study.

### Clinical features and CT image acquisition

We obtained clinical data by reviewing the patients’ hospital records. We recorded the patients’ age, sex, and smoking history. Data also included the results of a pulmonary function test (PFT). CT scans were obtained with 128‐detector‐row scanners (750HD, General Electric, Milwaukee, WI, USA; Somatom Definition, AS, Siemens Medical Solution, Forchheim, Germany). Technical parameters were as follows: detector collimation, 1–1.25 mm; beam pitch, 1; rotation time, 0.8–1 seconds; tube voltage, 120 kVp; tube current, 200–300 mA; 1 mm section spacing; and 512 × 512 pixel resolution. Images were reconstructed using a high spatial frequency (bone) algorithm with a slice thickness of 1.25 or 2 mm. All CT scans were recorded at the end of full inspiration.

### CT image analysis

**Figure 1:**
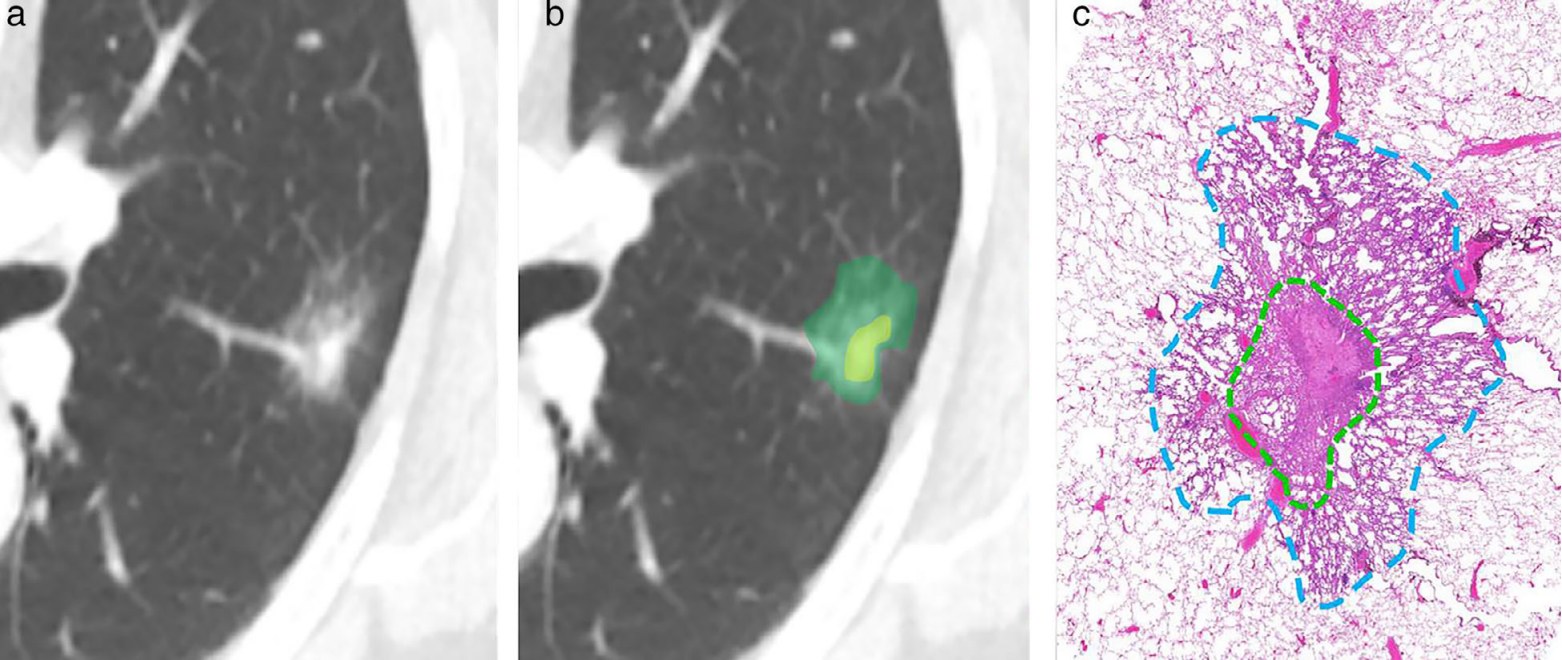
(**a** and **b**), representative computed tomography images showing measurement of whole tumor size (green) and sold tumor size (yellow) of part‐solid nodule. (**c**), Blue dotted line shows whole pathologic size, and green dotted line represents invasive tumor component.

Independent measurements were conducted by two chest radiologists (J.K.L. and K.M.S.) who were unaware of the patients’ clinical information. The longest diameter was measured with an electronic caliper using the lung window setting on multiplanar reformatted CT (W:1600; L:‐500). Each reader measured the largest diameters of the whole tumor (WTsize) and solid component (STsize) on representative images (Fig 1). When a tumor contained multiple solid components, each reader measured the size of the single largest solid component. We defined the ground‐glass opacity (GGO) ratio using the following formula: GGO ratio = (1 − [STsize/WTsize]) × 100 (%). In addition, two observers, in independent sessions, classified the nodules by morphology as subsolid or solid and recorded the presence or absence of visually detected emphysema on TSCT. Clinical T‐stages were assigned based on the size of the solid portion on TSCT. When there was discordance in determining presence of emphysema and solid component between two observers, the final assessment was made by consensus. The WTsize and STsize were determined by the mean of the two observers in all cases.

### Emphysema evaluation and scoring

In order to calculated the lobar emphysema score, all chest CT scans were transferred to a workstation (Syngo.Via, Siemens Medical Solutions, Forchheim, Germany). Pulmo3Dversion was used for segmentation of both lungs into lobes and quantification of emphysema. All image processing were performed by a fellow radiologist (H.L.) who was blinded to the purpose of this study. Lobe segmentation was performed to allow automated delineation for each of the five lung lobes using automatic lobe segmentation algorithm. Attenuation of each voxel within segmented lungs was computed automatically. A lower attenuation threshold of −950 HU on high‐resolution CT is known to correlate best with morphological emphysema; hence, emphysema volume was calculated as the sum of voxels with attenuation below −950 HU. Lobar emphysema score was calculated by the ratio of the emphysema volume to each lobe volume where the LAC was located as well as total lung volume.

### Pathologic evaluation

Histologic evaluation was performed by a pathologist (J.Y.J., with 10 years of experience in pulmonary pathology). All resected specimens were fixed in an inflated state by injection with 10% buffered formalin through the bronchus prior to sectioning and tissue processing. Sliced tissues were embedded in paraffin, and the blocks were sectioned and stained with hematoxylin and eosin. The maximal longest pathologic diameter (Psize) as well as the pathologic invasive size (PIsize) were recorded by the pathologist (Fig 1). Pathologic diagnoses were based on 2015 World Health Organization (WHO) classification criteria.^10^ Pathologic T‐stages were based on the sizes of the invasive components on pathologic analysis, according to the eighth edition of the TNM classification for lung cancer.^3^, ^11^

### Statistical analysis

All statistical analyses were performed using Statistical Package for the Social Sciences (SPSS), version 20.0 (SPSS Inc., Chicago, IL, USA). All numeric values are expressed as mean (range) ± standard deviation (SD). The normality of distributions was assessed with the Kolmogorov‐Smirnov test. Interobserver agreements for WTsize and STsize on CT were calculated by interclass correlation coefficient (ICC), and the 95% confidence interval (CI) for the ICC was also estimated. For comparison of CT and pathologic diameters, we first calculated the size differences of whole tumor (WTSD) and solid tumor diameters (STSD) between radiologic CT and pathologic measurements, respectively. We also calculated the size difference ratio (SDratio) with the formula: SDratio = (WTsize – Psize)/WTsize x 100 (%). Mann‐Whitney U tests were used to compare WTSD and SDratio between the emphysema group and normal group, while categorical variables were compared using either the chi‐squared test or Fisher's exact test. All *P*‐values less than 0.05 were considered statistically significant.

## Results

### Patient demographics

Table 1 shows patient demographics and clinical information for all 268 nodules in the present study, with 60 nodules in the emphysema group (22.4%) and 208 nodules in the normal lung group (77.6%). The mean patient age was 64.1 years (range, 35–80 years). The majority of nodules were found in women (60.8%), and most nodules manifested as a part‐solid nodule (PSN) (209/268, 78.0%) on TSCT. Clinical T‐stages were cT1 in 224 nodules (T1a in 70, T1b in 90, T1c in 64); cT2 in 42 nodules (T2a in 37, T2b in five); and cT3 in two nodules. Of the 268 nodules, 216 were resected by lobectomy and 52 by sublobar resection (16 by segmentectomy and 36 by wedge resection). Pathologically, 52 of 268 were stage T1a, 104 were T1b, 72 were T1c, 34 were T2a, and six were T2b.

**Table 1:** Clinicoradiologic findings between emphysema and normal lung group

	Emphysema (*n* = 60)	Normal lung (*n* = 208)	Total (*n* = 268)	*P‐value*
Gender				<0.001
Female	7 (11.7)	156 (75.0)	163 (60.8)	
Male	53 (88.3)	52 (25.0)	105 (39.2)	
Age	66.8 ± 7.4	63.3 ± 10.1	64.08 ± 9.7	0.004
Smoker	47 (78.3)	29 (13.9)	76 (28.4)	<0.001
Smoking PYRs	31.8 ± 22.4	4.1 ± 11.1	10.3 ± 18.4	<0.001
EEV1	94.1 ± 19.4	106.5 ± 22.1	103.8 ± 22.1	<0.001
FEV1/FVC	67.0 ± 10.0	74.5 ± 6.9	72.8 ± 8.3	<0.001
Emphysema score	2.2 ± 5.0	0.1 ± 0.3	0.6 ± 2.5	0.002
Nodule morphology				0.546
NSN	11 (18.3)	48 (23.1)	59 (22.0)	
PSN	49 (81.7)	160 (76.9)	209 (78.0)	
WTsize (cm)	2.4 ± 0.9	2.5 ± 1.1	2.5 ± 1.1	0.547
STsize (cm)	1.8 ± 1.0	1.9 ± 1.2	1.9 ± 1.2	0.638
GGO ratio (%)	28.2 ± 23.2	28.3 ± 41.1	28.3 ± 37.8	0.980
Clinical T‐stage				0.769
T1a	15 (23.4)	55 (27.0)	70 (26.1)	
T1b	21 (32.8)	69 (33.8)	90 (33.6)	
T1c	14 (21.9)	50 (24.5)	64 (23.9)	
T2a	11 (17.2)	26 (12.7)	37 (13.8)	
T2b	2 (3.1)	3 (1.5)	5 (1.9)	
T3	1 (1.6)	1 (0.5)	2 (0.7)	

Means ± standard deviation, Unless otherwise indicated, data are number of patients, with percentage in parentheses.

PYRs, Pack‐years; FEV1, forced expiratory volume in 1 second; FVC, forced vital capacity; WTsize, whole tumor size; STsize, solid tumor size; GGO, ground glass opacity.

The proportion of men and smokers was significantly higher in the emphysema group than in the normal lung group. Age, smoking pack‐years (PYRs), and emphysema score were significantly higher in the emphysema group. PFT, forced expiratory volume in 1 second (FEV1) and FEV1/forced vital capacity (FVC) were significantly lower in the emphysema group than in the normal lung group. However, there were no significant differences in nodule morphology, WTsize, STsize, or clinical T‐stage between the emphysema and normal lung groups.

### Comparison of whole tumor size measurements determined by radiology and pathology

**Figure 2:**
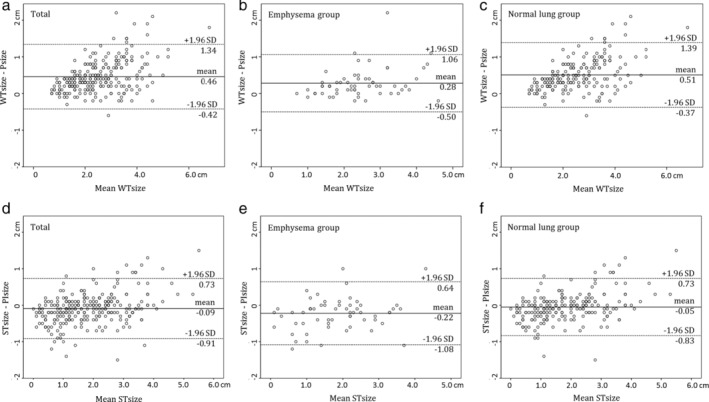
Bland‐Altman plots for radiologic whole and solid tumor measurements on TSCT recorded by two radiologists. (**a**,**b**,**c**) Bland‐Altman plots show agreement in whole tumor measurement between radiologic and pathologic assessment (**a**), in the emphysema (**b**) and normal lung group (**c**). (**d**,**e**,**f**) Bland‐Altman plots show agreement in invasive tumor measurement between radiologic and pathologic analysis (**d**), in the emphysema (**e**) and normal lung group (**f**).

The mean WTsize and Psize were 2.5 cm ± 1.1 (range, 0.7–6.9 cm) and 2.0 cm ± 0.9 (range, 0.5–5.0 cm), respectively. Interobserver agreement of the diameters measured by the two observers was excellent for WTsize (ICC = 0.968 [95% CI: 0.960–0.975]). Table 2 shows WTSD and SDratio between radiologic WTsize and Psize measurements according to clinicoradiologic findings. WTSD was significantly higher in the normal lung group *(P* = 0.001) and in PSNs *(P* < 0.001). In patients with a higher clinical T stage, WTSD was significantly higher *(P* < 0.001). SDratio was only significantly higher in patients with normal lungs (*P* = 0.015). Bland‐Altman plots with 95% CIs of the difference between WTsize and Psize according to the two groups are shown in Fig 2a‐c.

**Table 2:** Radiologic whole tumor and pathologic size measurements and differences according to clinicoradiologic findings

Feature	WTsize (cm)	Psize (cm)	WTSD	*P‐value*	SDratio	*P‐value*
No. of total subjects	2.5 ± 1.1	2.0 ± 0.9	0.5 ± 0.4		25.8 ± 26.7	
Gender						
Male	2.6 ± 1.0	2.2 ± 0.9	0.4 ± 0.4	0.164	23.1 ± 30.1	0.202
Female	2.4 ± 1.1	2.0 ± 0.9	0.5 ± 0.5		27.5 ± 24.3	
Lung status						
Normal	2.5 ± 1.1	2.0 ± 0.9	0.5 ± 0.4	0.001	28.3 ± 24.4	0.015
Emphysema	2.4 ± 0.9	2.1 ± 0.9	0.3 ± 0.3		17.0 ± 32.5	
Nodule morphology						
PSN	2.8 ± 1.0	2.3 ± 0.9	0.5 ± 0.5	<0.001	25.2 ± 26.6	0.511
NSN	1.6 ± 0.7	1.3 ± 0.5	0.3 ± 0.3		27.8 ± 27.5	
Clinical stage						
T1a	0.9 ± 0.1	0.7 ± 0.1	0.1 ± 0.1	<0.001	17.8 ± 22.5	0.510
T1b	1.6 ± 0.3	1.3 ± 0.3	0.3 ± 0.2		24.8 ± 26.4	
T1c	2.4 ± 0.3	2.0 ± 0.4	0.5 ± 0.4		29.8 ± 31.4	
T2a	3.4 ± 0.4	2.8 ± 0.5	0.6 ± 0.5		23.4 ± 19.7	
T2b	4.2 ± 0.4	3.5 ± 0.7	0.7 ± 0.7		24.8 ± 28.8	
T3	5.7 ± 0.9	4.3 ± 0.5	1.4 ± 0.8		32.1 ± 19.1	

Means ± standard deviation.

PSN, part‐solid nodule; NSN, nonsolid nodule; WTsize, whole tumor size; Psize, pathologic size; WTSD, whole tumor size difference; SDratio, size difference ratio.

### Comparison of solid tumor size measurements determined by radiology and pathology

The mean STsize and PIsize were 1.9 cm ± 1.2 (range, 0.0–5.5 cm) and 1.9 cm ± 1.0 (range, 0.1–5.0 cm), respectively. Interobserver agreement of the diameters measured by the two observers was excellent in STsize (ICC = 0.966 [95% CI: 0.957–0.974]). Table 3 shows STSD and SDratio between radiologic STsize and PIsize measurements according to clinicoradiologic findings. STSD was significantly higher in the emphysema group *(P* = 0.003) and in NSNs *(P* = 0.001). In patients with a lower clinical T stage, WTSD was significantly lower *(P* = 0.029). SDratio only significantly lower in patients with an NSN *(P* = 0.001) and a lower clinical T stage *(P* = 0.002). Bland‐Altman plots with 95% CIs of the difference between STsize and PIsize according to the two groups are shown in Fig 2d‐f.

**Table 3:** Radiologic solid and pathologic invasive size measurements and differences according to clinicoradiologic findings

Feature	STsize (cm)	PIsize	STSD	*P‐value*	SDratio	*P‐value*
No. of total subjects	1.9 ± 1.2	1.9 ± 1.0	0.5 ± 0.4		25.8 ± 26.7	
Gender						
Male	1.9 ± 1.1	2.1 ± 1.1	−0.1 ± 0.4	0.563	−3.1 ± 40.4	0.382
Female	1.8 ± 1.1	1.9 ± 1.0	−0.1 ± 0.4		−7.1 ± 27.8	
Lung status						
Normal	1.9 ± 1.1	1.9 ± 1.0	−0.1 ± 0.4	0.003	−3.7 ± 34.0	0.086
Emphysema	1.8 ± 1.0	2.0 ± 1.0	−0.2 ± 0.4		−12.0 ± 29.9	
Nodule morphology						
PSN	2.2 ± 1.0	2.2 ± 0.9	0.0 ± 0.4	0.001	−1.0 ± 27.8	0.001
NSN	0.7 ± 0.4	0.9 ± 0.5	−0.2 ± 0.4		−21.5 ± 44.8	
Clinical stage						
T1a	0.4 ± 0.3	0.6 ± 0.3	−0.2 ± 0.2	0.029	−33.3 ± 35.9	0.002
T1b	0.9 ± 0.4	1.1 ± 0.4	−0.2 ± 0.3		−10.7 ± 44.4	
T1c	1.7 ± 0.5	1.8 ± 0.5	−0.1 ± 0.4		−2.3 ± 29.1	
T2a	2.8 ± 0.6	2.8 ± 0.6	0.0 ± 0.4		0.2 ± 17.0	
T2b	3.5 ± 0.5	3.5 ± 0.7	0.0 ± 0.7		3.4 ± 22.1	
T3	4.6 ± 0.9	4.3 ± 0.5	0.3 ± 0.8		7.4 ± 19.2	

Means ± standard deviation.

PSN, part‐solid nodule; NSN, nonsolid nodule; STsize, solid tumor size; PIsize, pathologic invasive size; STSD, solid tumor size difference; SDratio, size difference ratio.

Changes in pathologic T‐stage with respect to clinical T‐stage between the emphysema and normal lung group are summarized in Table 4. A total of 44 of the 268 nodules were upstaged, as were 16 of 60 in the emphysema group and 28 of 208 in the normal lung group. Overall, 37 of 60 nodules in the emphysema group were concordant between clinical and pathologic T staging, compared to 159 of 208 nodules in the normal group (Table 4).

**Table 4:** Changes in pathologic T‐stage with respect to clinical T‐stage between emphysema and normal lung groups

	Emphysema group (*n* = 60)						Normal lung group (*n* = 208)							
Stage	cT1a (*n* = 14)	cT1b (*n* = 20)	cT1c (*n* = 18)	cT2a (*n* = 7)	cT2b (*n* = 1)	Total	cT1a (*n* = 56)	cT1b (*n* = 70)	cT1c (*n* = 46)	cT2a (*n* = 30)	cT2b (*n* = 4)	cT3 (*n* = 2)	Total	*P‐value*
pT1a (*n* = 52)	6	4	0	0	0	10	38	4	0	0	0	0	42	
pT1b (*n* = 104)	8	12	2	0	0	22	18	58	5	1	0	0	82	
pT1c (*n* = 72)	0	4	13	0	0	17	0	8	39	8	0	0	55	
pT2a (*n* = 34)	0	0	3	6	1	10	0	0	1	21	1	1	24	
pT2b (*n* = 6)	0	0	0	1	0	1	0	0	1	0	3	1	5	
Concordant	37/60 (61.7%)						159/208 (76.4%)							0.031
Discordant	23/60 (38.3%)						49/208 (23.6%)							

## Discussion

In the present study, we investigated the size discrepancy in the measurement of surgically resected subsolid LACs in TSCT and pathologic analysis and the impact of local emphysema on this discrepancy. We found that CT measurement of SSNs tended to overestimate the pathologic size of the whole tumor, and these differences were less prominent in the emphysema group than in the normal lung group. Interestingly, in measurement of the solid component of SSNs, the solid component in patients with emphysema was smaller than the invasive component on pathologic analysis, whereas the solid component on CT showed little difference from the invasive component on pathologic analysis in normal lung parenchyma. Furthermore, the concordance rate in T staging between CT and pathologic analysis was significantly lower in the emphysema group than in the normal lung group.

The overestimation of whole tumor size measurements on TSCT observed in our data is similar to the findings of previous studies.^5^, ^6^ Clinical tumor measurements are performed on TSCT images that are acquired at end‐inspiratory phase; thus, these measurements reflect the size of the tumor in an expanded lung. In contrast, pathologic measurements are obtained from deflated lung specimens after formalin fixation. Therefore, differences due to lung inflation may cause the discrepancy in tumor size between CT and pathologic measurements. In particular, it has been noted that estimates of size on gross examination of tumors that are predominantly lepidic (presenting as GGO on TSCT) were smaller than the actual tumor size of the LAC.^7^ Based on these observations, it may be speculated that the presence of emphysema, with histologic features of overinflated air spaces, may result in a smaller difference in whole tumor size between TSCT and pathology than in the normal lung. In our present study, WTSD was significantly higher in the normal lung group than in the emphysema group *(P* = 0.001). Moreover, in tumors with a higher T stage, WTSD was significantly higher *(P* < 0.001).

In LACs manifesting as SSNs, the solid component is the major determinant in predicting pathologic invasiveness, and the eighth edition of the TNM classification for lung cancer recommends that the clinical T stage of SSNs should be based on the size of the solid component on TSCT.^11^ In this regard, the most important finding of our study is that the solid component was smaller than the invasive component on pathologic analysis in the emphysema group; in contrast, there was little difference in TSCT and pathologic analysis in the normal lung group *(P* = 0.003). The explanation for this difference is again speculative. The presence of emphysema may decrease lung attenuation around the tumor, which may decrease the density of the entire SSN or the appearance of multiple solid components, resulting in a decrease in the final measurement of the solid component. Moreover, measuring the solid component can be affected by underlying lung attenuation because the distinction between the ground glass and solid components is presented as a small difference in the attenuation coefficient.

In the present study, an association was observed with downward‐migration in T staging in the emphysema group for 26.7% (16/60) of nodules, while downward‐migration occurred in 13.5% (28/208) of nodules in the normal lung group. In addition, concordance rates between clinical T staging based on CT and pathologic T staging were 61.7% in the emphysema and 76.4% in the normal lung groups, respectively *(P* = 0.023). Pathologically, lepidic growth in LAC is frequently underestimated on gross examination, and reproducibility of the distinction between invasive and lepidic patterns among pathologists varies widely, with only moderate interobserver agreement (kappa value, 0.55) in typical cases, and poor agreement (kappa value, 0.08) in difficult cases.^12^ Moreover, the pathological specimen is cut along the longest tumor dimension, and pathological T staging is primarily determined using two‐dimensional linear measurements. Therefore, the relative proportion of solid and ground glass components on TSCT may provide supplementary information to obtain an accurate impression of the PIsize in cases of discrepancies between the clinical and pathological tumor measurements. In addition, our findings regarding the effect of emphysema on tumor measurement in SSNs also may aid in interpreting these discrepancies.

The present study has several limitations. First, the small number of nodules analyzed and the retrospective nature of the analysis at a single institution may have affected our results. Second, we only evaluated LACs with pathologic size less than 5 cm, so these data may not apply to more advanced stages of LAC. Third, the number of pathologic T1 stage nodules in our study was relatively large compared to the number of pathologic T2 nodules, and among 228 nodules with pathologic T1 stage, 59 appeared as NSNs on TSCT. These size imbalances may have affected the discrepancy in STsize measurement in radiologic and pathologic analysis. Finally, CT measurements were based on dimensions obtained from the CT scan after deep inspiration, while pathologic measurements were made in the deflated state after surgical resection. Although we injected 10% formalin through the bronchus to prevent shrinkage and to minimize these measurement errors, these differences may have affected the size discrepancy.

The clinical implications of our study are twofold. First, WTsize of SSNs tended to overestimate the Psize, and these differences were less prominent in the emphysema group than in the normal lung group. Second, CT measurement of STsize was smaller than PIsize, and STsize for the normal group was better correlated with PIsize than that observed for the emphysema group. Accordingly, the concordance rate in T staging between CT and pathologic analysis was better correlated in patients with normal lungs than in those with emphysema.

In conclusion, the presence of emphysema was associated with the underestimation of solid tumor measurement on TSCT, which resulted in a higher discordance in T staging in TSCT and pathologic analysis for SSNs. These findings may be useful for the interpretation of discrepancies between clinical and pathological T staging of subsolid LACs in patients with emphysema.

## Disclosure

No authors report any conflict of interest.
